# Detection and antibiogram profile of diarrheagenic *Escherichia coli* isolated from two abattoir settings in northwest Ethiopia: a one health perspective

**DOI:** 10.1186/s42522-024-00102-y

**Published:** 2024-05-06

**Authors:** Solomon Lulie Abey, Mersha Teka, Abebe Belete Bitew, Wassie Molla, Mebrat Ejo, Gashaw Getaneh Dagnaw, Takele Adugna, Seleshe Nigatu, Bemrew Admassu Mengistu, Mebrie Zemene Kinde, Adugna Berju, Mequanint Addisu Belete, Wudu Temesgen, Shimelis Dagnachew, Tesfaye Sisay Tesema

**Affiliations:** 1 Department of Veterinary Pathobiology, College of Veterinary Medicine and Animal Sciences, University of Gondar, Gondar, P.O. Box 196, Ethiopia; 2 Department of Veterinary Epidemiology and Public Health, College of Veterinary Medicine and Animal Sciences, University of Gondar, Gondar, P.O. Box 196, Ethiopia; 3 Vaccine and Diagnostics Research & Development Division, Armauer Hansen Research Institute (AHRI), 1005, Addis Ababa, Ethiopia; 4 Department of Veterinary Biomedical Sciences, College of Veterinary Medicine and Animal Sciences, University of Gondar, Gondar, P.O. Box 196, Ethiopia; 5 Department of Veterinary Clinical Medicine, College of Veterinary Medicine and Animal Sciences, University of Gondar, Gondar, P.O. Box 196, Ethiopia; 6 Institute of Biotechnology, Addis Ababa University, Addis Ababa, Ethiopia; 7 Department of Veterinary Laboratory Technology, College of Agriculture and Natural Resource, Debre Markos University, Debre Markos, Ethiopia; 8 International Livestock Research Institute (ILRI), Addis Ababa, Ethiopia

**Keywords:** Abattoir setting, Antibiogram profile, Carcasses, Diarrheagenic E. Coli, Virulence genes

## Abstract

**Background:**

Diarrheagenic *Escherichia coli* (*E. coli*) is a zoonotic pathogen that contaminates abattoir workers, slaughter environments, slaughter equipment, and carcasses during abattoir processing. Infection with *E. coli* is associated with the consumption of contaminated food and water, and it is a potential threat to the health and welfare of both humans and animals. Hence, this study aimed to detect diarrheagenic *E. coli* and assess its antibiogram profile in two abattoir settings, in one health lens.

**Methods:**

A cross-sectional study in one health approach was conducted from December 2020 to June 2021. A total of 384 samples from abattoir workers’ hands, carcasses, knives, cattle feces, abattoir water and effluents were collected. Bacterial culture and biochemical tests were conducted to isolate *E. coli*, while conventional polymerase chain reaction was performed to identify virulence genes. The antibiogram of diarrheagenic *E. coli* was tested against nine antimicrobials using the Kirby Bauer disk diffusion method.

**Results:**

A total of 115 (29.95%) *E. coli* were isolated from the 384 samples, and from these isolates, about 17 (14.8%) were confirmed to be diarrheagenic *E. coli* (DEC). Among the DEC pathotypes, nine (52.94%), five (29.4%), and three (17.65%) were Shiga toxin-producing, enterohemorrhagic, and enterotoxigenic *E. coli*, respectively. While 14 (82.35%) DEC isolates harbored the *stx2* gene, five (29.41%) the *eae* gene, five (29.41%) the *hlyA* gene and three (17.65%) harbored the *st* gene. All the DEC isolates were resistant to erythromycin and vancomycin; whereas, they were susceptible to ampicillin, nalidixic acid and norfloxacin. Furthermore, 64.7% of DEC isolates showed resistance to both ceftazidime and kanamycin and 88.24% of the isolates showed multidrug resistance.

**Conclusion:**

This study detected DEC isolates having different virulence genes, which showed single and multiple antimicrobial resistance. Given the existing poor hygienic and sanitary practices along the abattoir-to-table food chain, coupled with the habit of raw meat consumption, this result indicates a potential public and animal health risk from the pathogen and antimicrobial resistance.

## Introduction

Foodborne pathogens are among the leading causes of illness and death worldwide [1], especially in developing countries, as the result of improper food management systems and inadequate food chain regulations [2]. Animal products such as milk, meat, eggs, fish, and their byproducts are typically regarded as high-risk commodities because they are suitable media for microbial invasion and growth [3].

Animal-origin foods have been linked to a number of harmful bacteria that affect the health and welfare of both humans and animals, i.e., having zoonotic importance [4]. The main bacterial pathogens usually found associated with animal-origin foods, but are not limited to: *Escherichia coli* (*E. coli*), *Staphylococcus aureus, Salmonella, Campylobacter*, and *Listeria monocytogenes* [5].

*Escherichia coli* is an enteric gram-negative, rod-shaped, facultatively anaerobic bacteria under the genus *Escherichia* that contains motile bacilli that fall into the family *Enterobacteriaceae* in the order *Enterobacterales* [6]. It is the most prevalent bacteria colonizing an infant’s digestive system after birth, and the host benefits from it for the balance of its life [7]. It is used as the main indicator during the evaluation of food contamination through faecal examination [8]. In addition, *E. coli* is an important zoonotic pathogen that can be linked to infectious diseases in animals and humans [9].

The *E. coli* consists of pathogenic groups and nonpathogenic commensals. Generally, the majority of nonpathogenic *E. coli* strains are not harmful, but there is a report that they have developed new virulence genes through horizontal gene transfer [6, 10]. Pathogenic *E. coli* consists of two groups namely, diarrheagenic *E. coli* (DEC) and extraintestinal pathogenic *E. coli* [11].

The DEC group consists of different strains, which includes enterotoxigenic *E. coli* (ETEC), enterohemorrhagic *E. coli* (EHEC), enteroinvasive *E. coli* (EIEC), enteropathogenic *E. coli* (EPEC), enteroaggregative *E. coli* (EAEC) and diffusely adherent *E. coli* (DAEC) [12]. The DEC group is known for its public health significance worldwide, since in most cases, it leads to diarrhea [13]. Infection is primarily associated with the consumption of contaminated food and water [14]. Several diarrheal outbreaks have been associated with the consumption of meat or meat products [15], and meat is contaminated by DEC in abattoirs at the time of processing [9].

The pathogenesis of DEC is associated with virulence factors [12]. Hence, each DEC has specific virulence genes responsible for coding virulence factors that interfere with the host’s physiology [16]. Among the most important genes, Shiga toxin (*stx*) is associated with STEC, heat-stable enterotoxin (*st*) and heat-labile enterotoxin (*lt*) are associated with ETEC, while enteropathogenic *E. coli* (EPEC) strains possess an intimin gene (*eae*) and bundle forming pilli gene (*bfpA*) [12, 17].

Different antimicrobial agents are used for the treatment of *E. coli*-associated infections in both humans and animals [18]. However, many virulent strains are claimed to acquire antimicrobial resistance, contributing its share to the global health challenge [19]. Antimicrobial resistance, the silent pandemic, is increasingly being detected in pathogens isolated from food [12].

Although data regarding the contamination of carcasses by the DEC in abattoirs are scarce in Ethiopia in general, in particular in northwest Ethiopia, there are few reports on the magnitude, microbial loads and *E. coli* O157:H7 contamination of meat. Some of the reports were 4.9% at the Mojo export abattoir [20], 9.3% at the Jima municipal abattoir [21], 8.1% at the Bishoftu slaughterhouse [22] and 8.9% at the Bahir Dar city beef carcass [23]. In the northwest part of Ethiopia, though there are some studies that reported the detection of DEC isolates and antimicrobial resistance from apparently healthy slaughtered animals, butcher shops, and butchers, there is limited evidence on the detection of DEC isolates, the virulence genes, and antibiogram profile from one health perspective [23]. Thus, the study was gird with the aim to isolate, detect and assess antibiogram profiles of DEC from slaughtered cattle (feces and carcass), slaughter environment (abattoir water and effluent) and equipment (knives), and hand swabs from abattoir workers in Gondar ELFORA and Bahir Dar municipal abattoirs, northwest Ethiopia.

## Materials and methods

### Study area and period

**Fig. 1:**
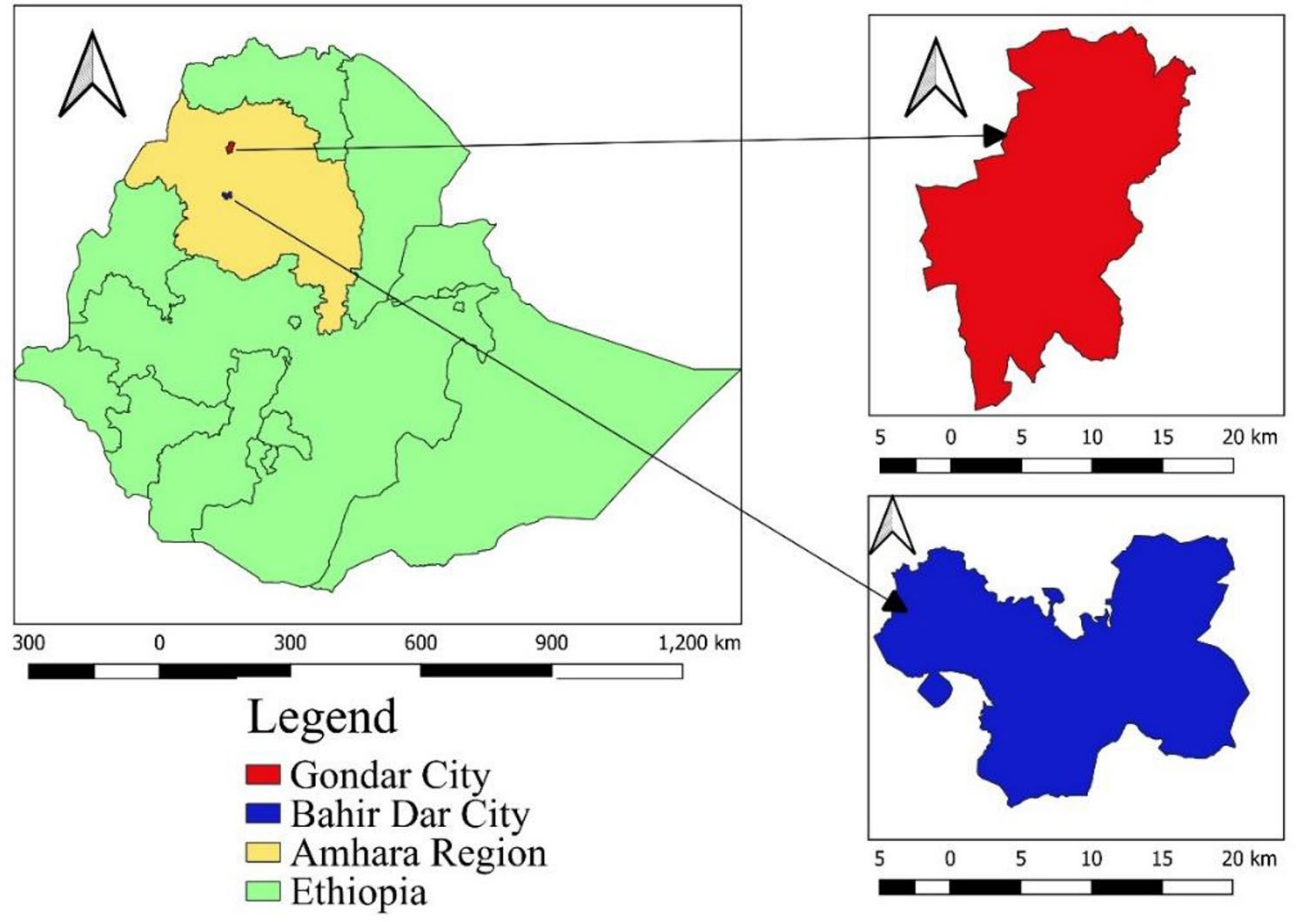
Geographical map of the study areas (prepared by QGIS 3.30 software)

The study was conducted at Gondar ELFORA and Bahir Dar municipal abattoirs from December 2020 to June 2021. Gondar ELFORA abattoir is located in Gondar city and is found in the Central Gondar zone of the Amhara region, north of Lake Tana and southwest of the Semen Mountains (Fig. 1). It is located at 12° 35’ 60.00"N latitude, 37°28’ 0.01” E longitude and 2,133 m above sea level. According to the Ethiopian Central Statistical Agency’s (CSA) 2007 national census population projection, the population of Gondar city in 2023 was projected at 429,278, with 200,299 men and 228,079 women [24]. Bahir Dar is the capital city of the Amhara region and one of Ethiopia’s most popular tourist destinations, having a range of attractions between Lake Tana and the Abay River. Bahir Dar city is located at 11°35’37.10"N and 37°23’26.77"E with an altitude of 1,820 m above sea level (Assefa *et al*., 2020), and the population of the city in 2023 was projected at 612,216 with 298,649 men and 313,567 women [24]. The livestock population of the Amhara National Regional State is estimated to be 17,262,804 cattle, 10,391,582 sheep, 7,045,305 goats and 19,060,608 poultry [25]. Gondar ELFORA and Bahir Dar municipal abattoirs provide slaughtering services to the communities and governmental or non-governmental organizations, including universities and the Dashen Brewery factory, to mention a few.

### Study design and sample type

A cross-sectional study from one health perspective was employed to collect study samples, including pooled swabs from carcasses, knives, slaughter workers’ hands, carcass washing water, cattle feces, and abattoir effluents.

### Sample size determination and sampling technique

Considering that there was no previous report that detected DEC in one health lens, and assuming that the number of cattle to be slaughtered from December 2020 to June 2021 is a finite population, the carcass sample size was determined using Arsham’s (2005) formula [26], *n* = 0.25/SE^2^, where n = sample size and SE = standard error, which is 0.05. The number of carcass swab samples for each abattoir was 100; pooled samples of knives, workers’ hands and water were collected separately during each visit so that 15 samples of each sample type were taken from each abattoir. Furthermore, 25 fecal samples from slaughtered cattle and 22 abattoir effluents were collected from each abattoir. Hence, a total of 384 samples in both abattoirs, which included 200 carcasses, 30 knives, 30 abattoir workers’ hands, 50 feces, 44 effluent and 30 water samples, were sampled (Table 1).

**Table 1:** Type and number of samples collected to detect DEC and assess its antimicrobial profile

Sample types	Unit/Sample	Number
Carcass surface	300 cm^2^	200
Abattoir worker’s hand	2 Hands	30
Knives	2 sides	30
Carcass wash water	25 ml	30
Cattle feces	10 gm	50
Abattoir effluent	200 ml	44
Total		384

To make the sampling easier and more representative, we used carcasses as a reference and started sampling apparently healthy animals presented to abattoirs for slaughter. Hence, a systematic random sampling technique was used to select apparently healthy animals presented for slaughter, (these animals were followed along the slaughtering procedure) from which carcass swab samples were collected. After assigning numbers for each animal, the sampling interval was determined by dividing the total number of animals to be slaughtered per sampling day by the needed sample size.

### Sample collection and transportation

Before slaughtering began, samples were collected from swabs of knives, slaughter workers’ hands and water. Knives were swabbed from the blade and handle surfaces, while for slaughter workers, the palms and fingers of both hands were swabbed horizontally and vertically. In both cases, pooled samples were collected to increase the chance of detection of the organism [3]. Sterile cotton tipped swabs soaked in buffered peptone water were used for these sample collections. After swabbing, the shaft of the swabs was broken and the cotton side of the swabs was kept in the sampling bottle. Twenty-five millilitres of water were taken from the tap immediately after the first stream was discarded and allowed to run for two to three minutes.

The neck, breast, thorax (lateral), abdomen (flank) and rump parts of the carcasses were swabbed and pooled after the carcasses were washed according to WHO guidelines [3]. A sterile cotton-tipped swab was prewetted in 10 ml buffered peptone water (Oxoid Ltd, Hampshire, England) to collect samples from each sampling region. Each sampling site was swabbed approximately 100 cm^2^, which is 10 cm horizontally and 10 cm vertically several times using separate sterile swabs [4]. The shafts were broken, and the cotton sides of the swabs were left in the sampling bottle once the rubbing was completed. In addition, 10 g of fecal samples were collected from the cecum of slaughtered animals. In addition, 200 ml of abattoir effluent was collected. Then, the samples were transported to the University of Gondar, Veterinary Microbiology laboratory under a cold chain and kept in a refrigerator at 4 °C until processing (i.e., bacterial culture and biochemical testing).

### Isolation and identification of *Escherichia coli*

Bacteriological loops were dipped in a bottle containing the original samples, and then a loop full of the sample was streaked primarily on MacConkey agar (Sigma Aldrich, United States) and incubated aerobically at 37 °C for 24 h. On MacConkey agar colonies with round shapes, smooth surfaces and pink color were suspected to be coliforms and were subcultured on Eosin-methylene blue (EMB) (HiMedia, India) agar. A single colony with a large, blue-black color and with or without a green metallic sheen on EMB agar [27] was isolated and further subcultured on nutrient agar (Himedia, India). Fresh colonies from nutrient agar were inoculated into test tubes containing sterile tryptone broth and incubated for 24 h at 37 °C followed by Indole, Methyl red, Voges-Proskauer and Citrate (IMVIC) and Triple sugar iron agar (TSI) tests [28, 29]. Finally, the bacterial isolates were cultured with 800 µl of tryptone soya broth (TSB) and incubated for 24 h at 37 °C and 35% glycerol was added. Then, the cultured samples were stored at -20 °C for further molecular characterization and antibiogram testing [30].

### DNA extraction

The preserved isolates were refreshed on EMB agar and a single colony was subcultured in TSB and incubated at 37 °C overnight [28]. Two ml of fresh culture from TSB culture was taken in an Eppendorf tube and centrifuged at 10,000 revolutions per minute (RPM) for 4 min. The supernatant was discarded, the cells were pelleted by adding 2 ml of culture broth and centrifuged at 10,000 RPM for 4 min, and the supernatant was discarded again. Then 100 µl of nuclease-free water was added for washing, dissolved, and centrifuged again at 10,000 RPM for 4 min and the supernatant was discarded. It was pelleted by adding 100 µl nuclease-free water, dissolved and heated at 100 °C for 10 min by heat block, followed by placing in deep freeze for 30 min [13]. Following these steps, the samples were boiled at 100 °C for 10 min, deep frozen for 5 min and centrifuged at 10,000 RPM for 10 min. Then, all the supernatant was separated and taken as the deoxyribonucleic acid (DNA) [31].

### Molecular detection of virulence genes

The conventional polymerase chain reaction (PCR) assay was used to detect *E. coli* virulence genes using specific primers (Table 2). Each PCR assay was performed in 25 µl final volume containing nuclease-free water, PCR buffer (Himedia, 2017), 0.35 millimolar (mM) of each dNTP (Himedia; India, 2017), specific forward and reverse primers (Bioneer; South Korea, 2017), Taq DNA polymerase enzyme (Delta Biotechnology) and DNA template (Table 3). The DNA samples carrying the relevant virulence genes served as positive controls in each reaction, while the negative controls were prepared from nuclease-free water that was used as a DNA template.

**Table 2:** Primers used for amplification of specific regions of the virulence genes of *E. coli*

Target gene	Primer code	Sequence (5’→3’)	Amplified products (bp)	References
*eae*	EAE1	F: AAACAGGTGAAACTGTTGCC	490	[32]
	EAE2	R: CTCTGCAGATTAACCTCTGC		
*stx2*	EVT1	F: CAACACTGGATGATCTCAGC	350	[33]
	EVT2	R: CCCCCTCAACTGCTAATA		
*hlyA*	EHCF F	F: ACGATGTGGTTTATTCTGGA	167	[34]
	EHCF R	R: CTTCACGTCACCATACATAT		
*bfpA*	BFPAF	F: AATGGTGCTTGCGCTTGCTGC	324	[35]
	BFPAR	R: GCCGCTTTATCCAACCTGGTA		
*st*	ST1	F: TTTATT TCT GTA TTG TCT T	294	[36]
	ST2	R: GCAGGATTACAACACAATTC		
*lt*	LT1	F: GGCGACAGATTATACCGTGC	696	[37]
	LT2	R: CCGAATTCTGTTATATATGTC		

Key: *eae* = intimin, *stx2* = shiga toxin2, *hlyA* = hemolysin gene, *bfpA* = bundle forming pilli, *st* = heat-stable enterotoxin, *lt* = heat-labile enterotoxin, F = forward primer, R = reverse primer, bp = base pair

**Table 3:** Preparation of the PCR mixture for each targeted gene

PCR component	Amount of PCR component (µl)		
	stx2 and eae	st and lt	bfpA and hlyA
Buffer	2.5	2.5	2.5
MgCl_2_	1.5	2	1.5
Nuclease free water	14.5	13	15
dNTP	1	1	1
Forward primer	1	1	0.5
Reverse primer	1	1	0.5
Taq DNA polymerase	0.5	0.5	0.5
DNA template	3	4	3.5
Total	25	25	25

Amplification was carried out with an initial denaturation temperature of 95 °C for 3 min followed by 35 cycles of each consisting of 40 s of denaturation, 40 s of annealing and 1 min of extension [28]. Denaturation and extension temperatures were 95 °C and 72 °C, respectively. Following 35 PCR cycles, each sample was subjected to final extension at 72 °C for 5 min. All amplifications were carried out in a Prima 96 plus thermal cycler (Himedia India).

### Agarose gel electrophoresis

Agarose gel (1.5%) was prepared by mixing 1.5 g of agarose powder with 100 ml of tris acetate ethylenediamine tetra-acetic acid (TAE) (40 mM Tris-HCl, 20mM acetate and 0.5 mM ethylenediamine tetra acetic acid) electrophoresis buffer, boiled in a hot oven for 2.5 min until the powder dissolved completely, and allowed to cool; then, 2.5 µl of ethidium bromide was added to the gel and mixed well. After the combs were placed onto the gel tray, the mixture was dispensed in the gel tray and allowed to solidify for 20 min. The tray with solidified gel was placed in the gel box containing 1×TAE electrophoresis buffer, and the combs were removed [4, 38].

Three microliters of loading dye were mixed with 10 µl of PCR product and loaded into the wells. A DNA ladder with 100 bp (Himedia; India, 2017) was run in parallel with PCR products to determine the size of the amplicons in bp. The gel electrophoresis was carried out by 110 millivolts for 60 min. The separated PCR products were visualized under ultra-violate transillumination (ultra-violate Tec, United Kingdom) [4, 38] and photographed in a gel documentation system and stored for further use (Bio-Rad; Germany).

### Classification of diarrheagenic *E. coli*

Classification of diarrheagenic *E. coli* was performed according to Kagambega et al. [18]. and Taha and Yasin [13].

### The antibiogram of diarrheagenic *Escherichia coli*

All DEC isolates were tested against ampicillin (10 µg), ceftazidime (30 µg), cefoxitin (30 µg), cotrimoxazole (25 µg), doxycycline (30 µg), erythromycin (15 µg), kanamycin (30 µg), vancomycin (30 µg), nalidixic acid (30 µg) and norfloxacin (10 µg) to investigate their antimicrobial resistance and susceptibility patterns using the Kirby Bauer disc diffusion method. Sterile Mueller Hinton agar-containing plates were used to perform the test. Pure DEC colonies from tryptone soya agar were taken using a sterile inoculating loop and added into sterile normal saline-containing test tubes. Then, the turbidity of the bacterial suspensions was compared and adjusted to 0.5 McFarland. Bacterial suspensions equal to that of 0.5 McFarland standard were inoculated onto Mueller Hinton agar plates by dipping sterile cotton swabs into the suspension, antimicrobial discs were dispensed using sterile forceps and then plates were incubated aerobically at 37 °C for 24 h. The zones of inhibition were measured using a caliper and then classified into resistant, intermediate and susceptible according to CLSI [19].

### Data management and analysis

Data collected from laboratory analysis were entered into a Microsoft Excel spreadsheet 2016. The proportions of DEC, virulence genes and antibiogram results were summarized by descriptive statistics. Fisher’s exact test was used to test the presence of a statistically significant association between abattoirs and the proportions of DEC, which was considered statistically significant when the p-value was less than 0.05. Statistical tools of the STATA version 17 software were used for data management.

## Results

### Frequency of *E. coli* Isolates

The frequency and distribution of *E. coli* among different sample sources are presented in Table 4. From the total of 384 samples, almost one-third (*n* = 115, 29.95%) were positive for *E. coli*, of which 33 (8.59%), 3 (0.78%), 4 (1.04%), 27 (7.03%), and 48 (12.5%) were from carcass, knives, abattoir workers’ hand, abattoir effluent and animal feces samples, respectively. From 50 slaughtered animal feces collected, 48 (96.00%) and from the 44 abattoir effluents, 30 (68.18%) were found positive for *E. coli* isolates. In addition, from 200 carcass swabs, 33 (16.50%), from 30 abattoir workers’ hands, four (13.3%), and from 30 cutting knives 3 (10%) were found positive for *E. coli* isolates. The proportion of *E. coli* isolates was 27.60% (53) at Gondar ELFORA, and 32.29% (62) at Bahir Dar municipal abattoir (Table 4).

**Table 4:** Frequencies of *E. coli* isolation by sample type and area

Sample source	Sample area	Total samples	No. of isolates by sample type and area (%)	Total No. of isolate by sample type (%)
Carcass	Gondar	100	15 (15.00)	33 (16.50)
	Bahir Dar	100	18 (18.00)	
Knives	Gondar	15	1 (6.70)	3 (10.00)
	Bahir Dar	15	2 (13.33)	
Hand swabs	Gondar	15	2 (13.33)	4 (13.33)
	Bahir Dar	15	2 (13.33)	
Water	Gondar	15	0 (0.00)	0 (0.00)
	Bahir Dar	15	0 (0.00)	
Effluent	Gondar	22	11 (50.00)	27 (61.36)
	Bahir Dar	22	16 (72.73)	
Cattle feces	Gondar	25	24 (96.00)	48 (96.00)
	Bahir Dar	25	24 (96.00)	
Subtotal	Gondar	192	53(27.60)	115(29.95)
	Bahir Dar	192	62 (32.29)	
Total		384	115(29.95)	115(29.95)

### Diarrheagenic *Escherichia coli* virulence genes and pathotypes

Of the 115 *E. coli* isolates, 17 (14.78%) were DEC, which have one or more of the virulence genes (Table 5). When it comes to the distribution of virulence genes across study locations, 12 were from Bahir Dar municipal and five were from Gonar ELFORA abattoir. The proportion of DEC pathotypes among the total *E. coli* isolates was found at 7.8% (9/115), 4.3% (5/115) and, 2.6% (3/115) for STEC, EHEC and ETEC, respectively (Table 6).

**Table 5:** The 17 diarrheagenic *E. coli* isolates with respect to virulence genes

Sample code	Abattoir	Sample type	Virulent gene				DEC
			stx2	eae	hlyA	st	
5	Gondar	Carcass	+	+	+	-	STEC
7	Gondar	Carcass	+	+	+	-	STEC
15	Gondar	Carcass	+	-	-	-	EHEC
17	Gondar	Carcass	+	-	-	-	EHEC
43	Gondar	Feces	+	-	-	-	STEC
56	Bahir Dar	Knives	+	-	-	-	STEC
58	Bahir Dar	Carcass	+	+	+	-	STEC
59	Bahir Dar	Carcass	+	+	+	-	STEC
60	Bahir Dar	Carcass	+	+	+	-	STEC
61	Bahir Dar	Carcass	+	-	-	-	STEC
62	Bahir Dar	Carcass	+	-	-	-	EHEC
64	Bahir Dar	Carcass	+	-	-	-	EHEC
67	Bahir Dar	Carcass	+	-	-	-	EHEC
69	Bahir Dar	Carcass	-	-	-	+	ETEC
71	Bahir Dar	Carcass	-	-	-	+	ETEC
75	Bahir Dar	Carcass	-	-	-	+	ETEC
106	Bahir Dar	Feces	+	-	-	-	STEC

DEC = diarrheagenic *E. coli*, STEC = Shiga toxin-producing *E. coli*, EHEC = enterohemorrhagic *E. coli*, EPEC = enteropathogenic *E. coli*, *stx2* = gene encoding Shiga toxin2, *stx* = Shiga toxin eae = gene encoding intimin, *hlyA* = hemolysin A gene, *st* = gene encoding heat-stable enterotoxin, + = positive and - = negative

**Table 6:** Frequencies of diarrheagenic *E. coli* pathotypes among the total *E. coli* isolates

Sample source	Sample Area	E. coli	DEC Pathotypes (%)			Sub-total (%)	Total (%)
			STEC	EHEC	ETEC		
Carcass	Gondar	15	2 (13.33)	2 (13.33)	0 (0.00)	4 (26.67)	14 (42.42)
	Bahir Dar	18	4 (22.22)	3 (16.67)	3 (16.67)	10 (55.56)	
Knives	Gondar	1	0 (0.00)	0 (0.00)	0 (0.00)	0 (0.00)	1(33.33)
	Bahir Dar	2	1(50.00)	0 (0.00)	0 (0.00)	1(50.00)	
Feces	Gondar	24	1(4.17)	0 (0.00)	0 (0.00)	1(4.17)	2(4.17)
	Bahir Dar	24	1(4.17)	0 (0.00)	0 (0.00)	1(4.17)	
Sub-total	Gondar	53	3(5.66)	2(3.77)	0 (0.00)	5(9.43)	17(14.78)
	Bahir Dar	62	6(9.68)	3(4.84)	3(4.84)	12(19.35)	
Total		115	9(7.83)	5(4.35)	3(2.61)	17(14.78)	17(14.78)

Key: *stx2* = Shiga toxin2 gene, *eae* = intimin gene, *hlyA* = hemolysin A gene and *st* = heat stable enterotoxin gene

Among the 17 DEC pathotypes, 82.35% (14/17) harbored the *stx2* gene, 29.41% (5/17) harbored the *eae* gene, 29.41% (5/17) harbored the *hlyA* gene and 17.65% (3/17) harbored *st* gene. The virulence gene detection rates from the total *E. coli* isolates were 12.17% (14/115) for the *stx2*, 4.3% (5/115) for the *eae*, 4.3% (5/115) for the *hlyA* and 2.6% (3/115) for the *st* genes (Table 7).

**Table 7:** Frequencies of diarrheagenic *E. coli* virulence genes based on sample source and abattoir

Sample source	Sample area	E. coli isolates	No. of virulence genes isolates (%)			
			stx2	Eae	hlyA	St
Carcass	Gondar	15	4 (26.70)	2 (13.33)	2 (13.33)	0
	Bahir Dar	18	7 (38.89)	3 (16.70)	3 (16.70)	3 (16.70)
Knives	Gondar	1	0 (0.00)	0 (0.00)	0 (0.00)	0 (0.00)
	Bahir Dar	2	1 (50.00)	0 (0.00)	0 (0.00)	0 (0.00)
Cattle	Gondar	24	1 (4.17)	0 (0.00)	0 (0.00)	0 (0.00)
feces	Bahir Dar	24	1 (4.17)	0 (0.00)	0 (0.00)	0 (0.00)
Sub -total	Gondar	53	5 (9.43)	2 (3.77)	2 (3.77)	0 (0.00)
	Bahir Dar	62	9 (14.52)	3 (4.84)	3 (4.84)	3 (4.84)
Total		115	14 (12.17)	5 (4.35)	5 (4.35)	3 (2.61)

Key: DEC = diarrhoeagenic *E. coli*, ETEC = enterotoxigenic *E. coli*, STEC = Shiga toxin producing *E. coli*, EHEC = enterohemorrhagic *E. coli*.

**Fig. 2:**
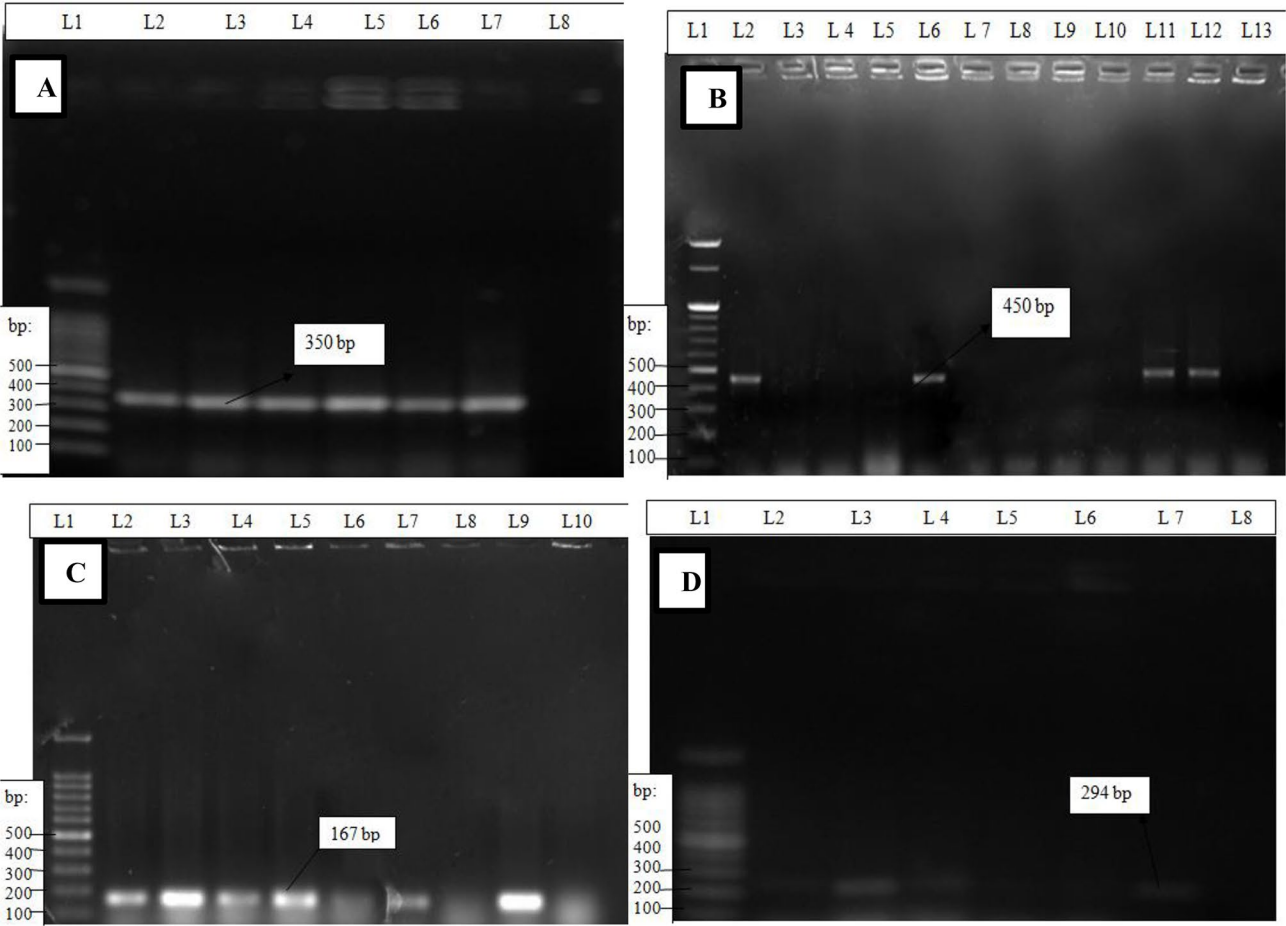
Representative gel electrophoresis results of PCR products for virulence genes. (**A**) Shiga toxin2 gene (350 bp); (**B**) intimin gene (450 bp); (**C**) hemolysin gene (167 bp) and (**D**) heat-stable enterotoxin gene (294 bp). L = lane, L1 = ladder (100 bp) (Himedia; India, 2017), bp = base pairs, L2 was a positive control for all genes and L8, L13, L10, and L8 were negative controls for the *stx2, eae, hlyA* and *st* genes, respectively

The detection rates of virulence genes from Gondar ELFORA abattoir *E. coli* isolates were 26.7% (4/15) *stx2*, 13.3% (2/15) *eae* and 13.3% (2/15) *hlyA* genes, and they were from the carcass samples, while only 4.2% (1/24) *stx2* gene was detected from animal feces samples. On the other hand, in the Bahir Dar municipal abattoir, 39% (7/18) *stx2*, 16.7% (3/18) *eae*, 16.7% (3/18) *hlyA* and 16.7% (3/18) *st* were isolated from carcass swabs isolates, 50% (1/2) *stx2* from knife swab isolates and 4.2% (1/24) *stx2* gene from feces sample isolates were found (Table 7). The *bfpA* and *lt* genes were not detected from any of the samples (Fig. 2).

Based on the DEC classification criteria, 52.94% (9/17), 29.41% (5/17) and 17.65% (3/17) of the DEC isolates were found to be STEC, EHEC and ETEC, respectively. Among sample types, the highest DEC pathotypes, 82.35% (14/17), were from carcasses followed by 11.76% (2/17) from feces and 5.88% (1/17) from knife swab samples. The highest number of DEC pathotypes, 70.59% (12/17), were identified from the Bahir Dar municipal abattoir, with 50% (6/12) STEC, 25% (3/12) EHEC and 25% (3/12) ETEC, whereas only 29.41% (5/17) DEC pathotypes were identified from the Gondar ELFORA abattoir, consisting of 60% (3/5) STEC and 40% (2/5) EHEC (Table 6).

The chi-square statistical analysis indicated that there was no significant difference between abattoirs in either *E. coli* (*P* = 0.316) or DEC (*P* = 0.135) isolation rates. There was a significant difference among sample types in both the *E. coli* (*P* = 0.000) and DEC (*P* = 0.000) isolation rates (Table 8).

**Table 8:** Association of *E. coli* and DEC isolation rate with sample source and types

	Sample source	*E. coli*		P-value	DEC		P-value
		Negative	Positive		Negative	Positive	
Abattoir	Gondar	139	53	0.316^a^	48	5	0.135^a^
	Bahir Dar	130	62		50	12	
Sample type	Carcass	83	7	0.000^a^*	19	14	0.000^b^*
	Feces	2	48		46	2	
	Carcass in-contact	83	7		6	1	
	Effluent	17	27		27	0	

Key: *statically significant, ^a^ P-value taken from Chi-square test, ^b^ P-value taken from Fisher’s exact test, DEC = Diarrheagenic *Escherichia coli*

### The antibiogram profile of diarrheagenic *Escherichia coli*

**Fig. 3:**
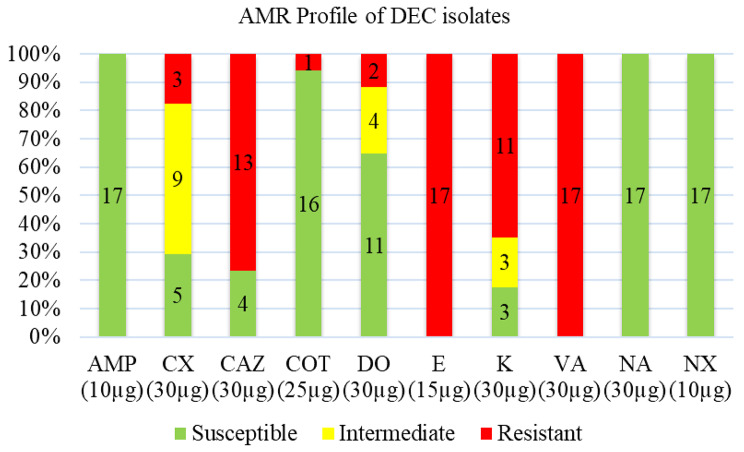
Antimicrobial susceptibility patterns of diarrheagenic *E. coli* isolates

All 17 DEC isolates were resistant to erythromycin and vancomycin, while 100% susceptibility was observed for ampicillin, nalidixic acid and norfloxacin. Against ceftazidime, kanamycin, cefoxitin, doxycycline and co-trimoxazole, 76.5%, 64.7%, 17.65%, 11.8% and 5.9% of the DEC isolates were resistant, respectively (Fig. 3). Multidrug resistance was observed in 82.4% (*n* = 14) of DEC isolates, of which two, one, eight and three isolates were resistant to six, five, four and three antimicrobials, respectively (Table 9).

**Table 9:** Multi-drug resistance profile of diarrheagenic *E. coli* isolates

MDR	Discs	DEC	DEC (%)
6	DO (30 µg), K (30 µg), E (15 µg), VA (30 µg), CX (30 µg), CAZ (30 µg)	STEC, ETEC	2 (11.8)
5	K (30 µg), E (15 µg), VA (30 µg), CX (30 µg), CAZ (30 µg)	STEC	1(5.9)
4	K (30 µg), E (15 µg), VA (30 µg), CAZ (30 µg)	STEC^b^, EHEC^b^, ETEC	8 (47.1)
	COT (25 µg), E (15 µg), VA (30 µg), CAZ (30 µg)	STEC	
3	E (15 µg), VA (30 µg), CAZ (30 µg)	ETEC, STEC	3 (17.65)
	K (30 µg), E (15 µg), VA (30 µg)	STEC	
2	E (15 µg), VA (30 µg)	EHEC^a^, STEC	3 (17.65)
>= 3			14 (82.4)

Key: AMP = ampicillin, CX = cefoxitin, CAZ = ceftazidime, COT = co-trimoxazole, DO = doxycycline, E = erythromycin, K = kanamycin, VA = vancomycin, NA = nalidixic acid and NX = norfloxacin, ETEC = enterotoxigenic *E. coli*, STEC = Shiga toxin-producing *E. coli*, EHEC = enterohemorrhagic *E. coli*. ^a^double, ^b^triple

## Discussion

In the current study, the overall proportion of *E. coli* was 29.94%. This finding was higher compared with the 22.2% prevalence reported by Haileselassie et al. [39], from the Mekelle municipal abattoir and 12.4% by Edget et al. [40], from the Dire Dawa municipal abattoir. From carcass swabs, the proportion of *E. coli* isolates was 16.5%, which was higher compared with the 7.5% *E. coli* proportion reported by Edget et al. [40]. and 10.9% reported by Hassen et al. [5]. at Dire Dawa and Asella abattoirs, respectively. In contrast, the current finding was lower than the reports of Haileselassie et al. [39], 22.2%, Edget et al. [40], 23.3% and Bersisa et al. [41], 35%, at Mekelle municipal, Haramaya University and Bishoftu abattoirs, respectively. The observed differences could arise from differences in the status of hygienic operations [17], geographical locations and slaughter processing conditions [14].

The overall prevalence of DEC, 14.9%, in this study is in line with the 11.6% and 13.6% isolation frequency of DEC reported by Taha and Yasin [13] from Iraq and Canizalez-Roman et al. [12]. from Mexico, respectively. However, the current report is lower than the reports of Kagambega et al. [18], 44%, from Burkina Faso and reported by Lee et al. [17], 35.5%, from Korea. However, it was higher than the report of Wang et al. [15], 6.3%, from meat samples in Japan and Rugeles et al. [16], 7.9%, from Colombia. The variation observed in DEC isolation might be attributed to differences in the epidemiology of the bacteria, the sanitation of the abattoirs, the sample types and the isolation techniques used.

At the Bahir Dar municipal abattoir, 55.60% of carcass swab isolates were DEC, which is in accordance with the 52% DEC isolation recorded by Taha and Yasin [13]. The significant DEC contamination of the carcass observed in this study might originate from fecal contamination during the animal slaughtering process [13]. The observed contamination of the carcasses with DEC in the current study would be an indicative of possible public and animal health risks from the two abattoirs [12].

The *stx2* gene detection rate in this study, 12.17%, was higher than the proportions of 0%, 4.3% and 6.3% previously reported by Taha and Yassin [13], Pizarro et al. [1]. and Wang et al. [15], respectively. The *eae* gene detection rate was also higher than the 0%, 1.85% and 3.3% reported from Pen Sylvenia [42], Argentina [1] and Argentina [43], respectively.

The proportion of *stx2* and *eae* genes detection in the current study looks higher than the other authors’ reports because these findings were compared against the number of *E. coli* isolates. However, the proportions decrease when compared relative to the whole samples (Table 6). The *st* gene detection proportion in this study was higher than the 1.7% detection rate from carcass samples in Mexico [12], but it was lower than the 13.5% detection from Japan [15].

The 7.8% (9/115) STEC pathotype reported in this study is in line with the 7.9%, 6.3%, and 9.5% STEC reported in Nairobi [9], Japan [15] and Iraq [13], respectively. However, it is lower than the 41.66% STEC recorded by Nehoya et al. [38]. from Namibia and the 25% STEC reported by Kagambega et al. [18]. from Burkina Faso. The difference in the results may be explained by the fact that samples from Burkina Faso were obtained from the open market, which increased the risk of contamination, while samples from Namibia were used to determine the STEC gene through a culturing system, which had the potential to obtain a high number of positive isolates.

According to Lee et al. [17]. in Korea, 22.6% of beef isolates from carcasses were found to be EHEC, which is higher than the current report, of 15.15%. The current finding was comparable with the 15% STEC reported from Mexico [12] but higher than the 9.5% EHEC reported from Iraq [13]. The disparities in contamination levels may be attributed to geographic variations in meat sources, slaughterhouse conditions, and procedures, such as the number, quantity, and length of time that samples were tested.

In the current study, a 9.09% ETEC was detected from carcass samples and this finding is comparable to the 9.8% ETEC detected in meat samples using the Biolog method. Wang et al. [15] and Odwar et al. [9]. reported a higher proportion than the current ones from Japan (13.5%) and Nairobi (60.3%), respectively. Lower ETEC detection proportions, 3.8% and 1.7% were reported by Tanih et al. [4]. from South Africa and Canizalez-Roman et al. [12]. from carcass samples in Mexico, respectively. ETEC is increasingly recognized as an important cause of foodborne illness since it has emerged as a major bacterial cause of diarrhea among travellers and children in the developing world [15].

The 4.17% STEC harboring the *stx2* gene recorded from fecal samples in the current study is comparable to the 5% STECS detection rate from slurry samples in Ouagadougou, Burkina Faso, but it is higher than the 0.58% detected in cattle feces and the 2.22% detected in manure in the composting process [2]. Geographical variations, abattoir conditions, and practices may be blamed for the variances in contamination levels.

All EHEC pathotypes possessed *stx2, eae* and *hlyA* genes together whereas STEC possessed stx2 genes and ETEC possessed the *st* gene only. The simultaneous presence of *stx2, eae* and *hlyA* genes enhances EHEC strain pathogenicity [13]. STEC and EHEC can cause severe foodborne disease; primary sources of outbreaks associated with these pathogens are raw or undercooked ground meat products, raw milk and fecal contamination of vegetables [4]. These pathogens are responsible for hemorrhagic colitis (HC) and hemolytic uremic syndrome (HUS) using their powerful toxins [12].

No *E. coli* was detected from water samples, which might be due to the smaller sample size or the laboratory technique used during isolation [41]. The slaughter workers’ hands were contaminated by *E. coli* (13.33% at both abattoirs) but none of the samples were positive for virulence genes. This finding was higher than the 0% and lower than the 50% of *E. coli* isolates reported at Dire Dawa and Haramaya University slaughterhouses, respectively [40].

The slaughter workers’ hand contamination might be due to poor personal hygiene, such as low frequency of hand washing, and absence of the habit of hand washing after toilet visits and after having contact with animals or farm visits. The knife swabs positivity to *E. coli* recorded in this study is lower than the 28% reported by Bersisa et al. [41]. from Knives swabs.

All of the DEC strains detected were resistant to erythromycin and vancomycin, while 100% susceptible to ampicillin, norfloxacin and nalidixic acid. This is in line with reports that all *E. coli* O157:H7 strains were 100% susceptible to norfloxacin and ampicillin [4, 20]. In another study, 8% resistance was observed for ampicillin [1]. In the current study, the DEC susceptibility to nalidixic acid was higher than the 72% susceptibility recorded by Haile et al., [21]and 17% susceptibility by Pizarro et al. [1]..

The next highest susceptibility of 93.3% was observed against co-trimoxazole which is lower than the 100% susceptibility observed by Haile et al. [21]. and Pizarro et al. [1]. against this drug. The current findings levels of resistance observed towards ceftazidime, 76.5% and kanamycin, 64.7%, were lower than 100% resistance for ceftazidime and 80% resistance for kanamycin which were reported by Abreham et al. [20]..

In the current study, 82.4% of DEC isolates showed multi-drug resistance. This is an alarming condition in which almost all antibiotics are resistant to pathogenic *E. coli* which might lead to difficulty in the treatment of human infection. The current finding is in disagreement with Gutema et al. [22], in which all *E. coli* O157 isolates were sensitive to the 14 antimicrobial drugs tested.

The reasons for the difference in the degree of susceptibility and resistance might be due to variability in the existence of resistance genes [12] and temporal and geographical differences between studies [18]. Food contamination with antibiotic-resistant bacteria can be a major threat to the public. Furthermore, the transfer of these resistant bacteria to humans has significant public health implications by increasing the number of foodborne illnesses [4].

The limitations of this study are butcher shops, restaurants and backyard slaughter systems were not included because of insufficient resources.

## Conclusion

The study confirmed the presence of DEC isolates, having different virulence genes. The *stx2* gene was found to be the most frequently isolated virulence gene and STEC had the highest isolated DEC pathotype. Most of the isolates were resistant to one or more commonly used antibiotics such as erythromycin, and vancomycin and multidrug resistance stands as an issue in this finding and it is a signal for a serious public health threat. Hence, an intervention with one health approach is crucial to mitigate the problem. Further research is needed to identify the possible human, animal or environmental origins and route of contamination at all stages of carcass processing in abattoirs and meat supply chains.

## Data Availability

Data are available and shared from the corresponding author upon reasonable request.

## Abbreviations

CLSI: Clinical Laboratory Standard Institute
DAEC: diffusely adherent *Escherichia coli*
DEC: Diarrheagenic *Escherichia coli*
*eae*: intimin gene
EAEC: Enteroaggregative *Escherichia coli*
*E. coli*: *Escherichia coli*
EHEC: Enterohemorrhagic *Escherichia coli*
EIEC: Enter invasive *Escherichia coli*
EPEC: Enteropathogenic *Escherichia coli*
ETEC: Enterotoxigenic *Escherichia coli*
*hlyA*: hemolysin A gene
PCR: Polymerase Chain Reaction
*st*: Heat-stable enterotoxin gene
*stx2*: Shiga toxin2 gene
